# Stakeholder’s response to COVID-19 using Protective Action Decision Model: Perception of Saudi citizens

**DOI:** 10.4102/jamba.v17i1.1886

**Published:** 2025-06-05

**Authors:** Praveen K. Maghelal, Michael Lindell, Hassan Taibah, Sudha Arlikatti

**Affiliations:** 1 Faculty of Resilience, Rabdan Academy, Abu Dhabi, United Arab Emirates; 2 Department of Urban Design and Planning, University of Washington, Seattle, Washington, United States; 3 Department of Public Administration, Faculty of Economics and Management, King Abdulaziz University, Jeddah, Saudi Arabia; 4 Amrita School for Sustainable Futures, Amrita Vishwa Vidyapeetham, Kollam, India

**Keywords:** COVID-19, protective action executed, perceived protective action efficacy, stakeholder perceptions, Saudi Arabia

## Abstract

Saudi citizens’ perceptions of community stakeholders involved with coronavirus disease 2019 (COVID-19) were assessed using the Protective Action Decision Model (PADM) model. Responses of 329 employees and students of King Abdulaziz University were analysed for their perceptions and protective actions taken in response to the risk of contracted COVID-19. Respondents rated the effectiveness in protecting themselves from COVID-19 by implementing six protective actions: staying home, physical distancing, cleaning surfaces, washing hands, using hand sanitiser and wearing a mask. They also reported the extent to which they had executed those protective actions. In addition, they rated six stakeholders: the Ministry of Health, government officials, news media, social media, peers (friends and relatives), and immediate family members on four characteristics. The extent to which each could be trusted, was responsible for their protection, had the knowledge needed to deal with the pandemic, and were relied upon for COVID-19 information. The results showed that perceived response efficacy is the strongest predictor of protective action implementation, and perceptions of the Ministry of Health are the best predictor of perceived response efficacy. These results have important practical implications that government agencies should address in future pandemics.

**Contribution:**

There is limited understanding about the Saudi citizens’ perception of stakeholders, especially with regard to the protective actions taken in response to COVID-19. Using the Protective Action Decision Model (PADM), this study provides insights into effectiveness of stakeholders and protective action in Saudi Arabia.

## Introduction

The Kingdom of Saudi Arabia (KSA) is vulnerable to several natural and man-made disasters (Jaziri & Miralam 2021). The recent onset of one of the worst health disasters of the decade tested the risk management strategies of countries world over. The disaster risk management strategies, as listed by the UN International Strategy for Disaster Reduction (UNISDR 2009), lists three phases, which include risk assessment and preparedness in pre-phase, effective response during the disaster phase, and the resilience of communities’ post-disaster. The KSA has made Disaster Risk Reduction (DRR) one of its priorities to manage the disasters within the country (Jaziri & Miralam 2021).

The KSA had an important role in managing the coronavirus disease 2019 (COVID-19) pandemic because of its strategic location at the crossroads of three continents (Africa, Asia and Europe) and because it hosts Hajj, which is the largest annual pilgrimage to the two holiest cities in Islam (Makkah and Madinah). During the pandemic, the KSA developed and implemented a multi-sectoral plan to decrease the risk of spreading COVID-19 both domestically and internationally, as well as to limit its economic impacts (AlFattani et al. 2021). The KSA’s risk management strategy started on 27 February 2020, with the suspension of visitor entry for Umrah in Mecca, as well as by visitors travelling from coronavirus-hit states for any other purpose (English Al Arabiya 2020a). On 05 March, the KSA temporarily closed the Grand Mosque in Makkah and the Prophet’s Mosque in Madinah at night to sterilise potentially contaminated surfaces (Arab News 2020a). On 08 March, it suspended schools in all its regions and governorates (English Al Arabiya 2020b) and also suspended travel of its citizens and residents to nine countries having high infection rates (Garda 2020). In addition, the KSA instituted restrictions on internal travel that included a travel ban between cities and a nationwide night-time curfew (Naar 2020a; Taibah 2020). In response to the pandemic, King Abdulaziz University (KAU) adopted extreme COVID-19 exposure control measures starting from March 2020 as it transitioned all its classes to online learning and provided its faculty with the required e-learning resources (Bardesi & Garba 2022).

The KSA immediately enhanced its healthcare infrastructure by increasing its capacity for testing and treating COVID-19 patients (Abdulaziz et al. 2023; Adly et al. 2020) through its well-established universal e-health system (Global Health Saudi n.d.; Taibah, Arlikatti & DelGrosso 2020a; Taibah et al. 2020b). To reduce the pandemic’s economic impact, 142 COVID-19-related measures that cost more than 214 billion rials – US$57 billion – were also implemented (Alzahrani, Aljamaan & Al-Fakih 2020; Taibah 2020). These measures included suspending certain labour-related fines and duties on imported products (Alzahrani et al. 2020), instituting the payment of 60% of private sector wages (Farouk 2020), and enabling digital access to most public services (Farouk 2020; Naar 2020b). Moreover, on a global scale, the KSA hosted the G20 summit on 26 March, promoting health disasters, such as COVID-19 at the top of its agenda (Naar 2020b). These measures limited the reported COVID-19 cases in KSA to approximately 540 000, with around 522 000 (about 98%) of whom recovered or were discharged and 8399 – (about 2%) of whom died (Taibah 2020; Worldometers n.d.). Thus, the KSA’s disaster risk management measures were successful in limiting the country’s fatality rate to less than 1% of all confirmed cases (Arab News 2020b).

It is evident that the KSA’s policies played a crucial role in limiting the risk of the spread of the virus, protecting public health and minimising economic impacts (Alonazi & Altuwaijri 2022; Sayed 2021). Nonetheless, questions remain regarding the ways in which these policies succeeded. To some degree, positive results were achieved by educating people about COVID-19 risk and protective actions. However, Taibah (2020) stated that not all Saudis had the same level of disaster awareness and protective response. This makes it important for health authorities to develop a better understanding of the risk communication process, especially the influence of different stakeholders on people’s implementation of official protective action recommendations. It is important to assess the effect of the KSA’s ‘We are all responsible’ campaign (Farouk 2020). One important aspect of this assessment is an examination of the influence of different stakeholders on people’s responses to COVID-19. To achieve this objective, this study examines Saudi citizens’ perceptions of different stakeholders’ protection responsibility, their hazard knowledge and trustworthiness and, thus, the degree of reliance on those stakeholders for COVID-19 information. Specifically, the perceptions and actions of students and employees of KAU were surveyed about the response to pandemic. As in most parts of the world, KAU adopted extreme COVID-19 exposure control measures starting from March 2020 as it transitioned all its classes to online learning and provided its faculty with the required e-learning resources (Bardesi & Garba 2022). Using online platform, this study assesses their perceptions of the efficacy of six different protective actions that were recommended by the KSA government and the degree to which they complied with those protective action recommendations.

## Stakeholders and their perceived characteristics

Understanding people’s perceptions of stakeholders relevant to disaster response is a complex and multifaceted issue. However, this understanding is critical for effective implementation and success of governments’ disaster risk management policies. For instance, Huang et al. (2022) highlighted the need for authentic community engagement, with a focus on health equity and mental health support, and Morrison et al. (2023) emphasised the importance of community engagement in decision-making, particularly in the context of restarting interventions. In addition, Cheetham et al. (2022) underscored the role of community-centred approaches with a focus on trust, leadership and lived experience. However, Gishe et al. (2020) provided a snapshot of the diverse responses to the pandemic, with some people demonstrating risk awareness and compliance with official protective action recommendations, while others violating recommended protocols.

These studies collectively highlight the importance of community engagement and the need to understand the community’s perception of a diverse range of stakeholders that are relevant to COVID-19 protective actions (Panneer et al. 2021). According to Drabek (1986), these can broadly be characterised as authorities (e.g. government agencies and non-governmental experts), news media and peers. Each type of stakeholder has a unique role to play in effectively addressing a pandemic’s challenges. Understanding the influence of all stakeholders is crucial because each stakeholder group contributes unique perspectives, resources and hazard knowledge, so collaboration among them is paramount in minimising crisis impacts (Loewenson et al. 2021).

People vary in their perceptions of the government’s response to the risks of COVID-19, with some viewing it as proactive and effective and others criticising it as slow or ineffective (see Martínez-Córdoba et al. 2021). These different perceptions can be influenced by factors such as reliance on the country’s healthcare system, government transparency, previous experiences with crisis and the level of trust in government authorities. For instance, Karokis-Mavrikos et al. (2022) found that Greeks varied in their perceptions of the government’s response to the risks of COVID-19, with some viewing it as proactive and effective and others criticising it as slow or ineffective. Furthermore, the perceived effectiveness of government agencies in managing the health disaster can also be influenced by the communication strategies they employed and the extent to which they incorporated scientific knowledge into their decision-making processes (Panneer et al. 2021).

In addition, some research has concluded that people’s responses to the pandemic have been affected by significant changes in society, such as the way that they perceive and consume media information (Fissi, Gori & Romolini 2022). According to this research, people’s response to the risk of contracting COVID-19 was influenced by the need for immediately accessible information, leading to increased reliance on social media platforms and a shift away from traditional news media sources (Fissi et al. 2022). However, reliance on social media can have both positive and negative consequences. On the positive side, social media allows for the rapid dissemination of information and facilitates communication among individuals. On the negative side, social media can propagate misinformation and conspiracy theories, which can fuel confusion and discourage compliance with authorities’ protective action recommendations (Desai et al. 2022).

In addition, family and peers play a crucial role in providing reliable information and guidance on COVID-19 prevention measures and vaccination. Moreover, they can support individuals’ physical and mental well-being by providing emotional support, help with practical needs such as grocery shopping or childcare, and a sense of community and connection during what is called ‘social distancing’ (Gayatri & Puspitasari 2022) but is actually *physical distancing* (National Academies of Sciences, Engineering, and Medicine 2021).

Social stakeholders vary in several characteristics that have been studied for many decades dating at least to Hovland, Janis and Kelley’s (1953) identification of expertise and trustworthiness as the primary components of an information source’s credibility – the degree to which messages from that source can be believed. Since that time, research on the psychology of persuasion has identified a number of secondary stakeholder characteristics (Gass & Seiter 2014), but research on risk communication has continued to confirm the importance of expertise and trustworthiness (Arlikatti, Lindell & Prater 2007; Arlikatti et al. 2019, 2022; Guo et al. 2022; Hyman et al. 2022; Sun et al. 2023; Wang, Wei & Shi 2018; Wei et al. 2018; Wu et al. 2020).

In addition, however, hazards research has identified two additional characteristics: protection responsibility (Arlikatti et al. 2007, 2019; Asgarizadeh Lamjiry & Gifford 2022; Guo et al. 2022; Hyman et al. 2022; Jackson 1981; Lindell & Whitney 2000; Murphy et al. 2018; Wang et al. 2018; Wei et al. 2018; Wu et al. 2017) and protection capability (also called institutional confidence or institutional trust) (Basolo et al. 2009; DeYoung & Peters 2016; Murphy et al. 2018; Wei et al. 2018). The findings from these studies are generally consistent with Drabek’s (1986) typology of information sources as authorities, news media and peers, but there are some stakeholders that do not fit neatly into these categories. For example, Hyman et al. (2022) reported that elected officials were more like news media than to government officials such as public health and disaster management authorities.

Godschalk et al. (1994) sought to explain the relative influence of different stakeholders by their *Onion Model*, which proposes that family and peers are the most credible information sources because of their stronger relationships and history. This model has been supported in some studies showing that stakeholders differ systematically in their perceived expertise, trustworthiness and protection responsibility. For example, Apatu et al. (2015) reported that self and peers rated higher on all three dimensions than news media which, in turn, were rated higher than officials. However, other research has found that authorities and news media are rated higher than self and family in expertise and trustworthiness and authorities are also considered to be high in protection responsibility (Jackson 1981). Moreover, peers are often (but not always) rated lower on all three characteristics than other stakeholders. Finally, these studies generally report significant positive relationships of stakeholder characteristics with protective action intentions and actual action (Arlikatti et al. 2007; Asgarizadeh Lamjiry & Gifford 2022; Guo et al. 2022; Hyman et al. 2022; Wang et al. 2018; Wei et al. 2018; Wu et al. 2017, 2020).

## Research objective

The fundamental objective of this study is to understand the effectiveness of the KSA’s COVID-19 policies in terms of their impact on people’s compliance with official protective action recommendations. Specifically, this study investigates the relationship of stakeholder perceptions with perceptions of protective action efficacy and the execution of COVID-19 protective actions. It is important to investigate these perceptions and actions because some studies have reported a lack of transparency in care, communication, and access to stakeholder information that affects community perceptions (Huang et al. 2022). While the issue of risk communication has been analysed for over many decades, little research has studied perceptions of stakeholders, especially attributes such as protection responsibility, hazard knowledge (or expertise), and trustworthiness and, thus, reliance on diverse information sources (Arlikatti et al. 2007; Kirschenbaum et al. 2017; Mărgărint et al. 2021). The results of this study can enhance community connectivity through community-centred approaches and mitigate the risk of negative effects on health and social inequalities in response to future health disasters such as COVID-19 (Cheetham et al. 2022). The COVID-19 pandemic has highlighted the importance of stakeholder collaboration and coordination in responding to a global health emergency (Panneer et al. 2021) and hence there is a need for research on the implementation of protective actions with regard to global health disasters, especially in the less investigated region of the Middle East. Thus, this study addresses four research questions regarding the KSA’s COVID-19 response:

RQ1. How do COVID-19 protective actions differ in their perceived efficacy and their actual implementation?

RQ2. How do stakeholders differ in their perceived characteristics?

RQ3. What are the best predictors of protective actions’ perceived efficacy and protective actions executed?

RQ4. Are the stakeholders closest to the respondents (family and peers) stronger determinants of protective actions executed than those that are remote (news media and government agencies), as predicted by the Onion Model?

## Research methods and design

### Survey administration

A convenience sample was collected from volunteering participants who were either employees or students at the KAU who were 18 years old or above and spoke either Arabic or English (the languages used in the questionnaire). The sample, which comprises 329 participants, was collected during May–July 2020, using an electronic questionnaire platform. An email with the purpose and objectives of the study, including a link to survey, was circulated inviting volunteers to respond to the survey. Follow-up invitations were sent asking volunteers to participate if they had not done so before or to ignore if they already responded to the survey.

### Measures

To measure protective action efficacy (Protective Action efficacy), respondents were asked ‘To what extent will each of the following personal protective actions protect you from coronavirus infection?’. The responses ranged from 1 (Not at all) to 5 (Very great extent) for six protective actions (see Joshi et al. 2015; e.g. Maghelal & Arlikatti 2025). To measure protective actions executed (PA executed), respondents used the same 5-point scale to rate the extent to which they had taken each protective action to protect themselves and their loved ones from COVID-19 infection. Respondents were also asked to report the extent to which they relied on each of six stakeholders and the extent to which each could be trusted, was responsible for their protection, and had the knowledge needed to deal with the pandemic. The stakeholders were Ministry of Health (MoH), government officials, news media, social media, peers (friends and relatives) and immediate family members. Finally, respondents were asked to report the number of household members in three categories: younger than 18 (< 18), between 18 and 65 (18–65), and older than 65 (> 65), and their age, gender, marital status, nationality, education, income and home ownership.

The PA efficacy ratings for the six protective actions were averaged to produce a scale with a high internal consistency reliability (α = 0.93). Similarly, PA executed ratings were averaged to produce a scale with α = 0.90. Moreover, ratings on each of the four stakeholder characteristics (reliance, protection responsibility, trustworthiness and hazard knowledge) were averaged to produce a scale score for each stakeholder. The resulting stakeholder scales had high levels of reliability for MoH (α = 0.90), government officials (GovOff α = 0.90), news media (α = 0.90), social media (α = 0.90), peers (α = 0.90) and family (α = 0.90). Finally, ratings on each of the six stakeholders were averaged to produce a scale score for each stakeholder characteristic. The resulting scales had high reliability for reliance (α = 0.80), protection responsibility (α = 0.80), trust (α = 0.82) and hazard knowledge (α = 0.86).

### Survey Data

A convenience sample was collected from volunteering participants who were either employees or students at the KAU, were 18 years old or above and spoke either Arabic or English (the languages used in the questionnaire). The sample, which comprised 329 participants, was collected between May and July 2020, using an electronic questionnaire platform. In response to the pandemic, KAU adopted extreme COVID-19 exposure control measures starting from March 2020 as it transitioned all its classes to online learning and provided its faculty with the required e-learning resources (Bardesi & Garba 2022).

As indicated in Table 1, the respondents’ ages ranged 18–70 with a mean of about 34 years, 53% of them were males (Female = 1, Male = 2), and over 90% of them were Saudi nationals. Over 50% owned their homes. Some households reported up to 15 members who are less than 18 years old, while some other households reported 15 individuals aged between 18 and 65 years old. Marital status averaged 1.56 (Married = 1, Single = 2) and the education level averaged 2.42 (Undergraduate degree = 2 and Graduate degree = 3), which is clearly because of the sampling of KAU employees and students. Income averaged *M* = 3.26 (3 = SR100K to 139K or USD 26K to 37K and 4 = SR140K to 179K or USD 37K to 48K).

**TABLE 1:** Descriptive statistics of variables to assess protective actions.

Variable	Definition	Mean	s.d.	Min	Max
PA executed	Protective Actions executed	4.36	0.83	1	5
PA efficacy	Perception: Protective action efficacy	4.38	0.81	1	5
Reliance†	Perception: Reliance on stakeholder	2.96	0.86	1	5
ProtResp†	Perception: Stakeholder protection responsibility	3.31	0.90	1	5
Trust†	Perception: Stakeholder trustworthiness	2.96	0.80	1	5
HazKnow†	Perception: Stakeholder hazard knowledge	3.34	0.88	1	5
Family‡	Source: Family members	3.14	0.98	1	5
Peers‡	Source: Friends and acquaintances	2.65	0.92	1	5
SocMed‡	Source: Social media	2.41	0.88	1	5
NewsMed‡	Source: News media	2.94	0.90	1	5
GovOff‡	Source: Government	3.33	1.01	1	5
MoH‡	Source: Ministry of Health	4.39	0.83	1	5
Age	Age of the respondent	33.89	11.90	18	70
HH < 18 years	No. of household members below 18	2.04	2.18	0	15
HH 18–65 years	No. of household members between 18–65	3.61	2.78	0	15
HH > 65 years	No. of household members over 65	0.46	1.01	0	8
Gender	Gender of the respondent	1.53	0.50	1	2
Marital status	Marital status of the respondent	1.56	0.54	1	3
Nationality	Nationality of the respondent	0.91	0.29	0	1
Education	Education level of the respondent	2.42	1.14	1	5
Income	Monthly income of the respondent	3.26	2.15	1	7
Home ownership	Home rented (0) or owned (1)	0.53	0.50	0	1

s.d., standard deviation; PA, protective actions; HH, household; No., number.

† , Average across all stakeholders;

‡ , Average of reliance, responsibility, trust, and hazard knowledge of the stakeholder.

### Analyses

Correlation analysis of PA executed, PA efficacy, stakeholder types, stakeholder attributes and demographic characteristics was followed by Ordinary Least Square regression analysis to identify the variables that are most important for predicting PA efficacy and PA executed. There were two analyses in the prediction of PA executed: the first without PA efficacy and the second with PA efficacy as a predictor variable.

In the analyses that follow, there are 30 statistical tests on mean differences, 231 on correlation coefficients, and 52 on regression coefficients, so the experiment-wise error rate is a concern (Ott & Longnecker 2015). Specifically, the expected number of false positive tests is False Positive = a x *n*, where *FP* is the number of false positive test results, a is the Type I error rate, and *n* is the number of statistical tests. If a = 0.05 and *n* = 313, then *FP* = 16. Benjamini and Hochberg (1995, see Glickman, Rao & Schultz 2014, for a more recent discussion) advocated that researchers specify, (1) a false discovery rate (*d*) for the entire study, (2) sort the *p*_*i*_ significance values for the individual tests in ascending order 1 £ *i* £ *n*, and (3) classify each *p*_*i*_ £ *d* x *i/n* as statistically significant (Glickman et al. 2014). In this study, the critical value of *p*_*i*_ = 0.01 so, for *N* = 329, a correlation *r* = 0.15 is statistically significant.

### Ethical considerations

Ethical clearance to conduct this study was obtained from the King Abdulaziz University Unit of Biomedical Ethics Research Committee (No. 248-20) for its adherence to the Declaration of Helsinki. All participants were in Jeddah, which is the second biggest city and considered the economic and tourism capital of Saudi Arabia (Jeddah Municipality n.d.) and were asked to provide an informed consent by clicking the accept link at the start of the survey. Responses were analysed using StataSE 18 to inquire the objective of the study.

## Results

### Outcome measures: PA efficacy and PA executed

**FIGURE 1:**
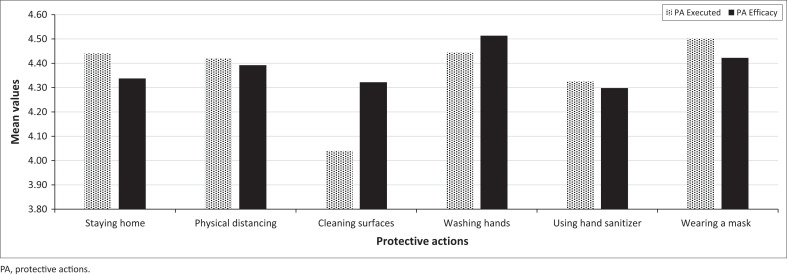
Perceived efficacy and reported execution of six protective actions.

Regarding RQ1 (How do COVID-19 protective actions differ in their perceived efficacy and their actual implementation?), Figure 1 shows the means of the six protective actions for both efficacy and execution by the respondent. The difference of mean test (*t*-test) for these actions indicates there is a statistically significant difference (*t*_*327*_ = 2.93, *p <* 0.01) in the ratings on PA efficacy for the actions with the highest (washing hands, *M* = 4.58) and lowest (using sanitiser, *M* = 4.29). Nonetheless, the high scale mean (*M* = 4.38) indicates that the respondents perceived all of the protective actions to have a high degree of efficacy.

In addition, a comparison of mean test (*t*-test) for the difference in PA executed between the levels of protective actions with the highest (masking, *M* = 4.50) and lowest (cleaning surfaces, *M* = 4.04) ratings was statistically significant (*t*_*327*_ = 5.40, *p <* 0.01). The high scale mean averaged over all six protective actions (*M* = 4.36) indicates that the respondents were highly compliant with the recommended protective actions.

### Stakeholder perceptions

**FIGURE 2:**
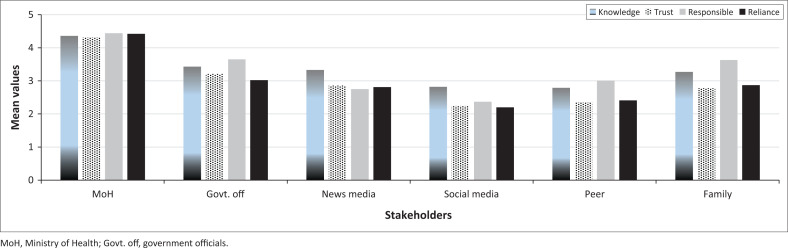
Mean ratings for perceived knowledge, trustworthiness, responsibility and reliance across six stakeholders.

Regarding RQ2 (How do stakeholders differ in their perceived characteristics?), Figure 2 shows that the respondents rated the MoH highest on all four characteristics (*M* = 4.39), followed by governmental officials (*M* = 3.33) and family (*M* = 3.14). Interestingly, news media and social media have higher ratings on knowledge than on the other characteristics – trustworthiness, protection responsibility and reliance. Peers have similar ratings to social media for all characteristics other than protection responsibility, for which the rating is much higher (*M* = 3.0 vs. 2.45, respectively). Family has higher ratings than peers on all four stakeholder characteristics. Although family ratings for protection responsibility are like those for government officials, family ratings for hazard knowledge, trustworthiness and reliance are comparable to those for news media. The rating of social media reliance is rather surprising because it received the lowest ratings for reliance of all stakeholders (*M* = 2.20), which was consistent with its low ratings of trustworthiness (*M* = 2.24).

### Correlation analyses

Table 2 (see Appendix 1 for full table) shows the correlations among the two dependent variables (PA executed and PA efficacy), the four stakeholder characteristics scale scores (reliance, protection responsibility, trustworthiness, hazard knowledge), the six stakeholder scale scores (family, peers, social media, news media, government officials, MoH), and the demographic variables (age, Household < 18 years, HH 18–65 years, HH > 65 years, gender, marital status, nationality, education, income and home ownership). The PA executed has a strong correlation with PA efficacy (*r* = 0.63), as well as statistically significant correlations with stakeholder characteristics (average correlation, $\bar{r}$ = 0.20; 0.17 £ *r* £ 0.24), and all the stakeholders except peers and social media ($\bar{r}$ = 0.19), but not with the demographic variables ($\bar{r}$ = 0.01). The PA efficacy has statistically significant correlations with all the stakeholder characteristics ($\bar{r}$ = 0.32) and stakeholders ($\bar{r}$ = 0.31), but not the demographic variables ($\bar{r}$ = −0.01).

**TABLE 2:** Correlation of PA executed, PA efficacy, stakeholders, and their attributes with demographic variables and protective actions.

No.	Variables	1	2	3	4	5	6	7	8	9	10	11	12	13	14	15	16	17	18	19	20	21	22
1	PA executed	1.00	-	-	-	-	-	-	-	-	-	-	-	-	-	-	-	-	-	-	-	-	-
2	PA efficacy	**0.63**	1.00	-	-	-	-	-	-	-	-	-	-	-	-	-	-	-	-	-	-	-	-
3	Reliance	**0.24**	0.32	1.00	-	-	-	-	-	-	-	-	-	-	-	-	-	-	-	-	-	-	-
4	Responsibility	**0.17**	0.44	0.47	1.00	-	-	-	-	-	-	-	-	-	-	-	-	-	-	-	-	-	-
5	Trust	**0.18**	0.26	0.78	0.38	1.00	-	-	-	-	-	-	-	-	-	-	-	-	-	-	-	-	-
6	Hazard knowledge	**0.22**	0.27	0.62	0.34	0.76	1.00	-	-	-	-	-	-	-	-	-	-	-	-	-	-	-	-
7	Family	**0.24**	0.29	0.66	0.47	0.68	0.73	1.00	-	-	-	-	-	-	-	-	-	-	-	-	-	-	-
8	Peers	0.12	0.24	0.73	0.50	0.71	0.68	0.81	1.00	-	-	-	-	-	-	-	-	-	-	-	-	-	-
9	Social media	0.01	0.16	0.67	0.55	0.68	0.59	0.50	0.63	1.00	-	-	-	-	-	-	-	-	-	-	-	-	-
10	News media	**0.24**	0.33	0.72	0.60	0.75	0.68	0.52	0.54	0.66	1.00	-	-	-	-	-	-	-	-	-	-	-	-
11	Gov. officials	**0.18**	0.25	0.68	0.52	0.68	0.56	0.37	0.41	0.43	0.63	1.00	-	-	-	-	-	-	-	-	-	-	-
12	Ministry of Health	**0.35**	**0.58**	**0.50**	**0.47**	**0.53**	**0.53**	**0.33**	**0.24**	**0.27**	**0.52**	**0.54**	1.00	-	-	-	-	-	-	-	-	-	-
13	Age	−0.01	−0.09	−0.03	−0.06	0.01	−0.10	−0.05	−0.02	−0.04	0.02	0.03	**−0.21**	1.00	-	-	-	-	-	-	-	-	-
14	HH < 18	0.03	0.09	0.11	0.09	0.04	0.03	0.10	**0.22**	0.14	−0.06	−0.02	0.00	−0.04	1.00	-	-	-	-	-	-	-	-
15	HH 18–65	0.12	0.13	0.05	0.04	0.03	0.07	0.09	0.08	0.03	−0.01	0.00	0.09	**−0.19**	**0.35**	1.00	-	-	-	-	-	-	-
16	HH > 65	−0.04	−0.05	−0.05	−0.03	−0.06	−0.04	−0.05	−0.01	−0.06	−0.02	−0.05	−0.05	**0.17**	**0.16**	0.13	1.00	-	-	-	-	-	-
17	Gender	−0.11	−0.14	0.06	−0.07	0.04	0.07	0.12	0.13	0.03	−0.06	−0.04	−0.02	**−0.17**	0.07	0.08	0.02	1.00	-	-	-	-	-
18	Marital status	−0.01	−0.04	−0.06	−0.06	−0.04	−0.10	−0.09	−0.03	−0.03	−0.04	−0.01	**−0.19**	**0.66**	0.10	**−0.31**	0.08	**−0.14**	1.00	-	-	-	-
19	Nationality	−0.02	0.03	−0.09	−0.10	−0.08	−0.04	−0.05	−0.08	−0.06	−0.12	**−0.16**	0.02	**−0.18**	**0.15**	**0.17**	0.10	−0.10	**−0.18**	1.00	-	-	-
20	Education	0.04	−0.08	−0.02	−0.07	0.01	−0.11	−0.10	−0.08	−0.06	0.03	0.06	**−0.15**	**0.70**	−0.04	**−0.22**	**0.16**	−0.14	**0.56**	**−0.26**	1.00	-	-
21	Income	0.10	0.05	0.01	−0.04	0.05	0.05	0.06	0.04	−0.06	−0.01	0.08	−0.02	**0.31**	**0.19**	0.09	0.10	0.07	**0.23**	0.01	**0.40**	1.00	-
22	Home ownership	0.00	0.00	0.00	0.00	−0.04	0.02	0.00	−0.02	0.00	0.00	−0.01	−0.02	−0.03	**0.16**	**0.22**	0.04	−0.12	−0.12	**0.34**	−0.06	0.11	1.00

Note: All correlations in bold are statistically significant at *p* < 0.01.

PA, protective actions; HH, household; Gov., government.

In addition, there are statistically significant correlations among the four stakeholder characteristics (average correlation, $\bar{r}$ = 0.56), and among the six stakeholders ($\bar{r}$ = 0.50), as well as between the four stakeholder attributes and the six stakeholders ($\bar{r}$ = 0.62). However, demographic variables have only five (out of 100) statistically significant correlations with the stakeholder characteristics and six stakeholders. Moreover, these correlations are very small in magnitude (average *r* = −0.10) and follow no apparent pattern.

Reliance has an extremely high correlation with trustworthiness (*r* = 0.78), indicating that people rely most strongly on their trust in stakeholders when deciding whether to rely upon them. Also, reliance has high correlations with peers (*r* = 0.73) and news media (*r* = 0.72) while trustworthiness has high correlations with hazard knowledge (*r* = 0.76), peers (*r* = 0.71) and news media (*r* = 0.75), and peers have a high correlation with family (*r* = 0.81).

There are high correlations among family, peers, and social media (a ‘peers’ cluster – average *r* = 0.65), and between government officials and Ministry of Health (an ‘authorities’ cluster – *r* = 0.54). In addition, there was a much lower correlation between these two clusters of stakeholders (average *r* = 0.28). News media are linked to both clusters, with an average *r* = 0.57 with peers and an average *r* = 0.58 with authorities.

Regarding RQ3 (What are the best predictors of protective actions’ perceived efficacy and protective actions executed?), Table 2 reports the results of correlation, which indicates that MoH (*r* = 0.58) and protection responsibility (*r* = 0.44) are the strongest predictors of PA efficacy, whereas PA efficacy is by far the strongest predictor of PA executed (*r* = 0.63) and MoH is the next strongest (*r* = 0.35).

There is mixed evidence regarding RQ4 – Are the stakeholders closest to the respondents (family and peers) stronger determinants of PA executed than those that are remote news media and government agencies, as predicted by the Onion Model. Specifically, family has a significant correlation with PA executed (*r* = 0.65), but peers and social media do not (*r* = 0.12 and 0.01, respectively). By contrast, MoH, government officials and news media all have significant correlations (*r* = 0.35, 0.18 and 0.24, respectively).

### Regression analyses

Three regression models examined the prediction of PA efficacy and PA executed from stakeholder characteristics, stakeholders and demographic characteristics, beginning with a transformation of the two dependent variables to normal distributions via a square root function. In addition, variables having extremely high correlations (*r* > 0.70) – three stakeholder attributes (reliance, trustworthiness and hazard knowledge) and one stakeholder (peers) – indicate an extremely high level of multicollinearity (see Vatcheva et al. 2016). So, following Cohen et al. (2003), they were omitted from the regression models. The models for PA efficacy, PA executed-I and PA executed-II all had statistically significant adjusted *R*^2^ = 41%, 18% and 43% respectively (Table 3 and Table 4).

**TABLE 3:** Prediction of PA efficacy and PA executed.

Determinants	PA efficacy			PA executed – I			PA executed – II		
	b	s.e.	β	b	s.e.	β	b	s.e.	β
Protection responsibility	**0.07**	**0.01**	**0.28**	0.00	0.02	−0.02	**−0.05**	**0.02**	**−0.21**
Family	0.02	0.01	0.11	**0.05**	**0.01**	**0.21**	**0.03**	**0.01**	**0.14**
Social media	−0.03	0.02	−0.13	**−0.07**	**0.02**	**−0.27**	**−0.05**	**0.02**	**−0.18**
News media	0.01	0.02	0.03	**0.06**	**0.02**	**0.22**	**0.05**	**0.02**	**0.20**
Government officials	**−0.04**	**0.01**	**−0.19**	−0.02	0.02	−0.07	0.01	0.01	0.05
Ministry of health	**0.14**	**0.02**	**0.54**	**0.08**	**0.02**	**0.28**	−0.02	0.02	−0.08
Age	0.00	0.00	−0.02	0.00	0.00	−0.06	0.00	0.00	−0.05
No. of < 18 years	0.00	0.01	0.04	0.00	0.01	0.02	0.00	0.01	−0.01
No. of 18 to 65 years	0.01	0.00	0.08	0.01	0.00	0.13	0.01	0.00	0.08
No. of over 65 years	−0.01	0.01	−0.04	−0.01	0.01	−0.05	−0.01	0.01	−0.03
Gender	−0.05	0.02	−0.11	−0.04	0.02	−0.10	−0.01	0.02	−0.02
Marital status	0.04	0.03	0.09	0.02	0.03	0.05	0.00	0.03	−0.01
Nationality	0.01	0.04	0.01	−0.01	0.05	−0.01	−0.01	0.04	−0.02
Education	0.00	0.01	−0.01	0.02	0.02	0.10	0.02	0.01	0.11
Income	0.01	0.01	0.06	0.01	0.01	0.05	0.00	0.01	0.02
Home ownership	−0.01	0.02	−0.03	−0.01	0.03	−0.03	0.00	0.02	−0.01
PA efficacy	-	-	-	-	-	-	**0.69**	**0.06**	**0.66**
Number of obs.	329.00	-	-	329.00	-	-	329.00	-	-
*df*	16.00	-	-	16.00	-	-	17.00	-	-
*R* ^2^	0.44	-	-	0.22	-	-	0.46	-	-
Adj. *R*^2^	0.41	-	-	0.18	-	-	0.43	-	-
*F*	15.38	-	-	5.35	-	-	15.73	-	-
Significance (*p*)	0.00	-	-	0.00	-	-	0.00	-	-

Note: All variables in bold are statistically significant at *p* ≤ 0.01.

PA, protective actions; s.e., standard error; obs, observations; no., number.

**TABLE 4:** Reduced model of PA efficacy and PA executed.

Determinants	PA efficacy			PA executed – I			PA executed – II		
	s.e.	b	β	s.e.	b	β	s.e.	b	β
Protection responsibility	0.07	0.01	0.28	-		-	−0.05	0.02	−0.21
Family	-	-	-	0.05	0.01	0.21	0.03	0.01	0.14
Social media	-	-	-	−0.07	0.02	−0.27	−0.05	0.02	−0.18
News media	-	-	-	0.06	0.02	0.22	0.05	0.02	0.20
Government officials	−0.04	0.01	−0.19	-	-	-	-	-	-
Ministry of Health	0.14	0.02	0.54	0.08	0.02	0.28	-	-	-
Age	-	-	-	-	-	-	-	-	-
No. of < 18 years	-	-	-	-	-	-	-	-	-
No. of 18 to 65 years	-	-	-	-	-	-	-	-	-
No. of over 65 years	-	-	-	-	-	-	-	-	-
Gender	-	-	-	-	-	-	-	-	-
Marital status	-	-	-	-	-	-	-	-	-
Nationality	-	-	-	-	-	-	-	-	-
Education	-	-	-	-	-	-	-	-	-
Income	-	-	-	-	-	-	-	-	-
Home ownership	-	-	-	-	-	-	-	-	-
PA efficacy	-	-	-	-	-		0.69	0.06	0.66
Number of obs.	329.00	-	-	329.00	-		329.00	-	-
*df*	16.00	-	-	16.00	-		17.00	-	-
*R* ^2^	0.44	-	-	0.22	-	-	0.46	-	-
Adj. *R*^2^	0.41	-	-	0.18	-	-	0.43	-	-
*F*	15.38	-	-	5.35	-	-	15.73	-	-
Significance (*p*)	0.00	-	-	0.00	-	-	0.00	-	-

Note: All variables in bold are statistically significant at *p* ≤ 0.01.

PA, protective actions; s.e., standard error; no., number; obs, observations.

Regarding RQ3 (What are the best predictors of protective actions’ perceived efficacy and actual executed?), Table 3 shows PA efficacy was significantly predicted by Ministry of Health (β = 0.54), protection responsibility (β = 0.28) and government officials (β = −0.19). The negative sign for government officials should be treated with some caution because this variable has a positive correlation with PA efficacy (*r* = 0.25), and conflicting signs for correlation and regression coefficients often indicate multicollinearity. Multicollinearity is especially likely because the perception of government officials has a moderately strong positive correlation with the perceptions of other stakeholders ($\bar{r}$ = 0.48). Consequently, the regression equation for PA efficacy was re-estimated with government officials deleted, which produced a negligible decrease in the variance accounted for (D adj. *R*^2^ = −0.01). Thus, perceptions of government officials have at most a trivial effect on PA efficacy.

The first PA executed model (PA executed – I), which included all variables except PA efficacy, shows that this variable is predicted by four stakeholders namely, MoH (β = 0.28), news media (β = 0.22), family (β = 0.21) and social media (β = −0.27). As was the case for the regression coefficient in the prediction of PA efficacy from government officials, the negative sign for social media in the prediction of PA executed should be treated with some caution because these variables have a nonsignificant correlation (*r* = 0.01). As was the case with the prediction of PA efficacy from government officials, multicollinearity is a plausible explanation because social media has strong positive correlations with the other stakeholders (average *r* = 0.50). Consequently, the regression equation for PA executed was re-estimated with social media deleted, which produced a negligible decrease in the equation fit (D adj. *R*^2^ = −0.03). Thus, perceptions of social media have at most, a trivial effect on PA executed.

The second PA executed model (PA executed – II), which added PA efficacy to the prediction equation, produced a substantial increase in adjusted *R*^2^ from 0.18 to 0.43. This model had significant coefficients for PA efficacy (β = 0.66), news media (β = 0.20), family (β = 0.14), protection responsibility (β = −0.21) and social media (β = −0.18). As was the case for the regression coefficient in the prediction of PA executed – I, the negative signs for protection responsibility and social media in PA executed – II should be treated with some caution because of these variables’ small (*r* = 0.17) and nonsignificant (*r* = 0.01) correlations, respectively. In this case, multicollinearity is a plausible explanation because the perception of protection responsibility also has strong positive correlations with the perceptions of other stakeholder attributes (average *r* = 0.40). Consequently, the regression equation for PA executed – II was re-estimated with protection responsibility and social media deleted, which produced a negligible decrease in equation fit (D adj. *R*^2^ = −0.04). Thus, perceptions of protection responsibility and social media have at most, trivial effects on PA executed.

The regression analyses also produced mixed evidence regarding RQ4 – Are the stakeholders closest to the respondents (family and peers) stronger determinants of PA executed than those that are remote (news media and government agencies.)? Consistent with the Godschalk et al. (1994) *Onion Model* proposing that family and peers are the most credible information sources, PA executed – I included family (a close stakeholder). Inconsistent with that model, however, the Ministry of Health and the news media (remote stakeholders) are also significant predictors of PA executed. Similarly, PA executed – II continued to include family and the news media, but not the Ministry of Health, as predictors of PA executed.

## Discussion

This study examined the influence of KSA residents’ perceptions of stakeholders and stakeholder characteristics on their perceptions of protective action efficacy and their execution of COVID-19 protective actions. Regarding RQ1 (How do COVID-19 protective actions differ in their perceived efficacy and their actual implementation?), similar to other studies (Kowalski & Black 2021; Nazione, Perrault & Pace 2021), the respondents reported high levels of protective action efficacy and protective action executed. All of the protective actions received high ratings of efficacy (overall *M* = 4.38), but hand washing was especially high (*M* = 4.51). Moreover, most of the protective actions received high ratings of actual implementation (overall *M* = 4.36), but cleaning surfaces was noticeably lower although still high (*M* = 4.32). This disparity between the high ratings of efficacy and the lower level of execution for cleaning surfaces can be explained in two ways. Firstly, the respondents might have been less concerned about the risks related to COVID-19 exposure by touching contaminated surfaces than by inhaling COVID-19 viruses. This possibility suggests that future research should assess people’s perceptions of different hazard exposure paths for respiratory infectious diseases, just as Lindell et al. (2015) did for water contamination.

Secondly, the Protective Action Decision Model (PADM) suggests that respondents might have considered theresource requirements of cleaning surfaces (Lindell 2018; Lindell & Perry 2012). Specifically, respondents might have believed that any reduction in COVID-19 exposure from cleaning surfaces was not worth the time and effort required to execute this protective action. To test this explanation, future research should examine people’s perceptions of each protective action’s *net benefit* (the hazard-related attributes – protection of persons and property, utility for other purposes) in relation to its resource requirements (cost, time/effort, knowledge/skill, tools/equipment, social cooperation). In addition, this research should examine the *incremental net benefit* for each protective action, given that other protective actions are being executed.

Regarding RQ2 (How do stakeholders differ in their perceived characteristics?), the respondents reported higher levels of reliance, trustworthiness, hazard knowledge and protection responsibility in the Ministry of Health than any other stakeholder. These results are consistent with the finding that source credibility is influenced by expert credentials and past history of job performance (Perry & Lindell 1990).

Ratings for government officials in general were lower than for the Ministry of Health and approximately the same as for the news media, but they were much higher than ratings for peers and social media. The finding that social media has lower ratings of trustworthiness than other stakeholders is consistent with the findings of Tayal and Bharathi (2021) but not Fissi et al. (2022). However, the rest of the results align with other studies (Algahtani et al. 2021; Almutairi et al. 2020), indicating that Saudis had very positive perceptions of the disaster management experts in the Ministry of Health, which produced high ratings of COVID-19 protective action efficacy. In turn, high ratings of protective action efficacy produced high levels of compliance with official protective action recommendations for limiting the risk of exposure and spread of COVID-19.

Consistent with the results of previous studies, protection responsibility exceeded knowledge about dealing with disasters for family (Arlikatti et al. 2007; Lindell & Whitney 2000) but, in this case, also for peers. In addition, family protection responsibility exceeded that of all stakeholders except the Ministry of Health. This is slightly different from the results of US earthquake studies, which found that family/self-protection responsibility exceeded that of all other stakeholders including government agencies. Also consistent with previous results, family knowledge of dealing with disasters was perceived to exceed that of peers (Arlikatti et al. 2007; Lindell & Whitney 2000). However, this pattern of perceived stakeholder characteristics differs from the respondents’ perceptions of protection. Finally, responsibility definitely has a positive effect on PA efficacy (*r* = 0.44; β = 0.28), but a weak correlation (*r* = 0.17) and a nonsignificant or possible negative effect on PA executed (β = −0.02 in Model I, β = −0.21 in Model II). As noted earlier, it is likely that the coefficient for protection responsibility in PA executed Model II is an artefact of multicollinearity, so the most reasonable conclusion is that the effect of protection responsibility on PA executed is completely mediated by its effect on PA efficacy.

## Practical implications and conclusions

Understanding community perceptions of various stakeholders, both governmental and non-governmental, is critical to the development of effective policies for response to health-related disasters. Thus, this study investigated KAU employees’ and students’ perceptions of six stakeholders, regarding their knowledge, trustworthiness, reliance and responsibility for protection from community from the risk of contracting COVID-19. Their responses indicate much more strongly positive perceptions of the Ministry of Health, government officials, news media and immediate family than of social media and peers regarding COVID-19 protective actions. News media and family appear to have direct effects on protective action execution, whereas the effect of the Ministry of Health appears to be mediated by its effect on perceptions of protective action efficacy.

Although this study provides important insights into stakeholder perceptions, it does have some limitations. One of these limitations is the non-representative sample of KSA citizens that was because of COVID-19 restrictions. Limiting responses to KAU employees and students, rather than the Saudi population in general, means that the data set is missing the stakeholder perceptions and COVID-19 responses of households with lower incomes and less education. However, the under-representation of those households would, if they were systematically different from the KAU sample, be expected to attenuate the variance of the variables in the model and, thus, underestimate the correlations (Nunnally & Bernstein 1994). Also, the data are cross-sectional, so it is not possible to determine if stakeholder perceptions caused the implementation of the protective actions or vice versa. Nonetheless, this study has four important practical implications that disaster management agencies, specifically the public health professionals, should address in any future pandemics.

### Reliance on official sources

Although there was high reliance on the Ministry of Health, this was less true for other governmental agencies. Thus, to the extent that citizens need to rely on other government agencies for disaster-related information (e.g. Ministry of Hajj and Umra, Ministry of Foreign Affairs, Ministry of Communication and Information Technology, Ministry of Civil Service), those agencies should work to ensure that the perceptions of their knowledge and trustworthiness about health-related disasters are commensurate with their perceived protection responsibility. This can be achieved by ensuring transparency in communication across all governmental bodies involved in managing the risks from other pandemics in future.

### Reliance on informal sources

Perceptions of social media and peers are distinctly different from those of the news media and government agencies in general, let alone the Ministry of Health. Although reliance on social media and peers is low, consistent with their low ratings of trustworthiness, these sources’ ratings of knowledge of health disasters are near the midpoint of the response scale. That is, the respondents apparently believed that these sources could provide some accurate information. This finding indicates that authorities should monitor social media to promptly detect misinformation and maintain their own social media accounts and disseminate accurate information.

### Reliance on the news media

Perceptions of the news media are similar to those of government agencies other than the Ministry of Health. Consequently, the news media should also engage in efforts to ensure transparency in communication about health-related disasters and protective actions. Considering the KSU is host to the two major pilgrimage sites of Islam, the media should also ensure information dissemination in multiple languages.

### Promoting protective actions through perceived efficacy

Government policies should aim to increase the adoption of officially recommended preventive actions by emphasising the efficacy of those actions in preventing risk of infection. In addition, however, it will be important to address the disaster-related resources of those protective actions, specifically their financial cost and requirements for Knowledge and skills, time and effort, tools and equipment and cooperation of others (Lindell & Perry 2012). In some cases, this will involve reducing misperceptions about those resource requirements, whereas in other cases, it will require government agencies to take actions that will reduce those resource requirements. As an example of reducing a protective action’s resource requirements, many workplaces installed hand sanitiser stations, so people did not need to provide their own bottles of hand sanitiser to reduce the risk of contracting COVID-19 in public areas.

Overall, these policy implications underscore the importance of a comprehensive and inclusive approach to managing a health disaster such as COVID-19. In addition to addressing people’s perceptions of risk of contracting COVID-19 and recommended protective actions, government agencies should consider risk perceptions of different societal stakeholders and their characteristics.

## Appendix 1

**TABLE 1-A1:** Correlation analysis.
